# Targeting of Non-Dominant Antigens as a Vaccine Strategy to Broaden T-Cell Responses during Chronic Viral Infection

**DOI:** 10.1371/journal.pone.0117242

**Published:** 2015-02-13

**Authors:** Peter J. Holst, Benjamin A. H. Jensen, Emeline Ragonnaud, Allan R. Thomsen, Jan P. Christensen

**Affiliations:** 1 Department of International Health, Immunology and Microbiology, University of Copenhagen, The Panum Institute, Copenhagen, Denmark; 2 Centre for Medical Parasitology, Department of International Health, Immunology and Microbiology, Faculty of Health and Medical Sciences, University of Copenhagen, Copenhagen, Denmark; University of Iowa, UNITED STATES

## Abstract

In this study, we compared adenoviral vaccine vectors with the capacity to induce equally potent immune responses against non-dominant and immunodominant epitopes of murine lymphocytic choriomeningitis virus (LCMV). Our results demonstrate that vaccination targeting non-dominant epitopes facilitates potent virus-induced T-cell responses against immunodominant epitopes during subsequent challenge with highly invasive virus. In contrast, when an immunodominant epitope was included in the vaccine, the T-cell response associated with viral challenge remained focussed on that epitope. Early after challenge with live virus, the CD8+ T cells specific for vaccine-encoded epitopes, displayed a phenotype typically associated with prolonged/persistent antigenic stimulation marked by high levels of KLRG-1, as compared to T cells reacting to epitopes not included in the vaccine. Notably, this association was lost over time in T cells specific for the dominant T cell epitopes, and these cells were fully capable of expanding in response to a new viral challenge. Overall, our data suggests a potential for broadening of the antiviral CD8+ T-cell response by selecting non-dominant antigens to be targeted by vaccination. In addition, our findings suggest that prior adenoviral vaccination is not likely to negatively impact the long-term and protective immune response induced and maintained by a vaccine-attenuated chronic viral infection.

## Introduction

Adenovirus based vaccines delivering the antigen linked to the MHC class II associated invariant chain (Ii) induce potent T-cell responses against antigens that are not normally very immunogenic [1–3]. Indeed, fusion of the glycoprotein of lymphocytic choriomeningitis virus (LCMV) to Ii markedly improves adenovector-induced protective efficacy against acute and chronic infections, whereas effects of Ii fusion is much more subtle in the case of the immunodominant NP protein. Overall, we have been able to induce responses which were quantitatively similar against antigens that are highly different in their intrinsic immunogenicity, and both GP and NP targeted vaccines were able to control LCMV infection in the acute phase [3]. Exploiting this fact, we decided to study the consequences of vaccine antigen selection on the immune responses evolving against vaccine encoded and non-vaccine encoded antigens during the chronic phase of the subclinical infection induced in vaccinated mice challenged with highly invasive LCMV. An additional benefit of this strategy is that we can compare virus-specific, adenovector primed and non-primed responses in the same animals. Such studies are very important as a series of novel vaccine strategies, based on different viral antigen expression platforms, are being developed against the important chronic viral infections caused by HIV and HCV. Examples of such new vaccine approaches are the adenovector based vaccines involving rare human serotype prime-boost regimens tested by Dan Barouch and co-workers at Harvard [4,5], and the adenovector based strategies applied by Thomas Hanke and McMichael against HIV [6] and by Alfredo Nicosia and collaborators against HCV [7–9]. Generally, the vectors are used to target the most important T cell antigens during natural infection, and the immunization regimens apply potent vaccine vectors for which humans are largely immunologically naïve. The change in vector between the prime and the booster immunization allows for efficient transduction at both immunizations. Targeting the most dominant antigens may be a necessity for achieving relevant levels of acute viral control, but based on a range of publications in recent years, it comes at the risk of not only a narrowly focused T-cell response, but also of reduced functionality of the induced antiviral response in the long-term. Indeed, several studies have suggested that repeated antigenic stimulation may drive T cells into an effector memory (KLRG-1+/CD127+/-) state characterized by a high cytotoxic potential, but at the cost of reduced proliferative capacity, susceptibility to apoptosis, and poor control of systemic infection [10–12].

Targeting the most immunogenic antigens, however, is not the only option available. Using adenovectors expressing Ii linked non-dominant LCMV GP antigen, we can now show that efficient virus control may be obtained by targeting the intrinsically non-dominant GP antigen, and that this allows for a potent CD8 T cell response to be elicited by virus encoded dominant NP antigen during the chronic phase of the high-dose infection. In contrast, when mice were initially vaccinated using the dominant NP antigen, the subsequent virus elicited response remained focused on the major NP epitope. During the early period after virus challenge, we could confirm previously recorded observations regarding phenotypic changes in repeatedly stimulated T cells in those T cells primed by the vaccine and boosted by the virus infection (e.g. higher KLRG-1 expression [10,13]). However, these differences were not maintained one year after infection in the dominant specificities and, more importantly, at this time adoptive transfer experiments demonstrated that NP specific T cells proliferated equally well irrespectively of whether NP had been included in the original vaccine or not. Based on these results it is suggested that 1) the targeting of sub-dominant antigens can be applied to broaden responses against specificities not included in the vaccine, but also that 2) chronic low-grade antigen stimulation reduces the impact of the stimulation history. Broadening of vaccine and virus elicited immune responses offers theoretical advances against chronic and genetically unstable infections, as the vaccine elicited non-dominant antigen response, which had initially enabled virus control was highly stable over time, and would potentially prevent viral recrudescence if the virus should later mutate its primary antigenic determinants.

## Materials and Methods

### Mice

All experimental procedures were approved by the national animal ethics committee (Dyreforsøgstilsynet) and performed according to national guidelines. C57BL/6 and BALB/c mice were obtained from Taconic M&B (Ry, Denmark), whereas B6.SJL (CD45.1) mice were the progeny of local breeder pairs originating from The Jackson Laboratory (Bar Harbor, ME). All mice enrolled in these studies were between 7–10 weeks old and housed in a specific pathogen free facility as validated by testing according to FELASA guideliness.

### Viral vectors and immunizations

Production of the IiGP, GP, IiNP and NP expressing replication defective adenoviral vectors used throughout this study has been described previously [3].The inserted antigens are derived from the LCMV Armstrong 53b strain. Infectious titers were determined using the Adeno-X rapid titer kit (CLONTECH) on infected HEK293 cells, and immunizations were performed by injection of 2x10^7^ infectious units into the right hind foot pad.

### Viruses

LCMV clones 13 and Armstrong 53b were originally obtained from M. B. A. Oldstone (The Scripps Research Institute, La Jolla, CA) and subsequently propagated in-house. The Traub strain of LCMV was produced and stored as previously described [14]. For infections, mice were put in a restrainer and injected via a tail vein with 10^6^ pfu of LCMV clone 13, 10^4^ pfu of LCMV Armstrong 53b or 200 pfu of LCMV Traub strain, each dose diluted in a volume of 0.3 ml PBS.

### Adoptive cell transfer

Spleens from unvaccinated, Ad-IiGP or Ad-IiNP vaccinated C57BL/6 (CD45.2) mice that had been challenged 1 year earlier with LCMV clone 13 were harvested and single cell suspension was obtained by pressing the spleens through a 70 μm disposable filter (Millipore). The cells from 5 mice of each experimental group were pooled and washed twice by gentle centrifugation. Numbers of epitope-specific cells were determined by standard tetramer staining followed by flow cytometric analysis. Spleen suspensions containing 10^4^ cells of relevant specificity (i.e. 10^4^ NP396 cells or 10^4^ GP33, GP34 and GP276 combined) per recipient were diluted in PBS and injected i.v. into the tail veins of B6.SJL (CD45.1) mice.

### Vaccinia challenge

The day after cell transfer, recipients were injected with vaccinia virus expressing either LCMV GP or NP. Recombinant vaccinia virus expressing GP of LCMV (VV-GP) was originally obtained from Dr. D.H.L. Bishop (Oxford University, Oxford, U.K.) via Annette Oxenius (ETH, Zürich, Switzerland) and grown on CV-1 cells at low multiplicity of infection; quantification was performed as described previously [1]. Mice to be infected with vaccinia virus were injected with 2x10^6^ pfu in 200 μl intraperitoneally (i.p.). Six days post challenge, the spleen from each mouse was isolated, and the number of epitope-specific donor CD8+ T cells was determined by tetramer staining and flow cytometry. The fold expansion was calculated by comparison with naïve mice receiving the adoptive cell transfer, but no vaccinia challenge. Donor cells were separated from recipient cells based on the expression of CD45.1 and CD45.2.

### Organ virus titers

Organs were homogenized in PBS to make 10% organ suspensions, and viral titers were determined using an immune focus assay as previously described [15].

### Spleen cell preparations and flow cytometry

Single cell suspensions of splenocytes were obtained by pressing the organs through a fine steel mesh, followed by centrifugation and resuspension in RPMI cell culture media. The cells were then incubated with 0.1 μg/ml of relevant peptide for 6 hours as described previously except that the cells were incubated without monensin for the first hour [16]. Functional epitope specific CD8+ T cell responses were enumerated by surface staining for CD8 (Pe/Cy5.5 or Pacific Blue), CD44 (APC/Cy7), CD19 or B220 (PerCP/Cy5.5 and pacific blue respectively) and intracellular staining for IFN-γ (APC). Thus, cells enumerated in this study represent numbers of CD8+, CD44+, IFN-γ+ and CD19/B220- cells in the spleens of analyzed mice. Total numbers were calculated by multiplying the total number of cells in the spleens determined using an automated cell counter, and the percentage of specifically gated cells. Phenotypic analysis was performed by additional staining for the surface markers, KLRG-1 (PE) and CD127 (Pe-Cy7). All antibodies were purchased from Biolegend. Cell samples were run on a Becton-Dickinson LSRII FACS machine, and data analyses were performed using Flow Jo (Treestar) software.

### Statistical analyses

Weight curves were analyzed using two-tailed Unpaired T test whereas T cell responses were compared using the Mann-Whitney U test.

## Results

### Vaccines targeting LCMV GP and NP

**Fig 1:**
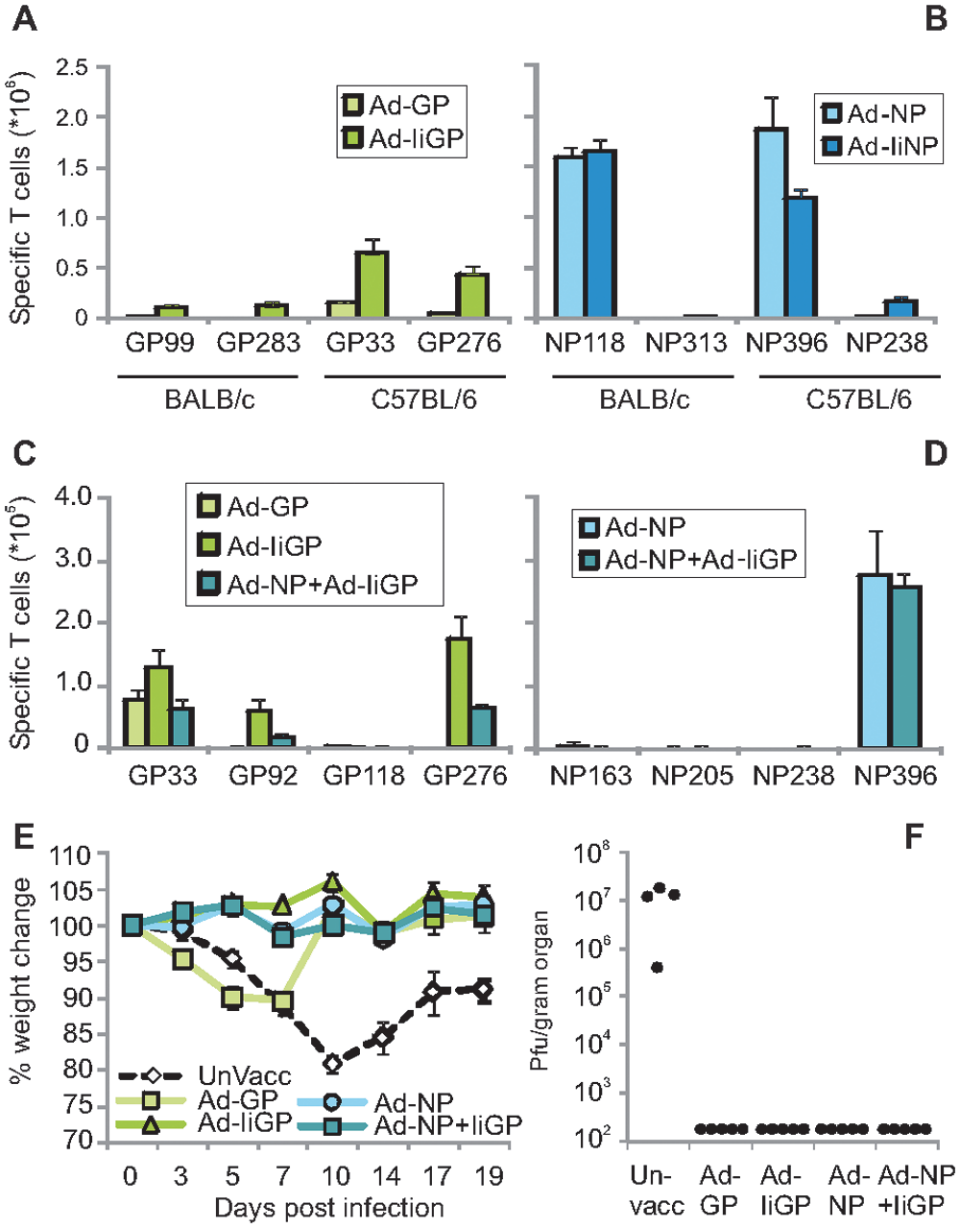
Immunogenicity of adenoviral vaccines in BALB/c and C57BL/6 mice and vaccine induced control of LCMV clone 13 infection. **a**) CD8+ T cell responses obtained 14 days post vaccination against GP derived H-2^d^ (left columns) and H-2^b^ (right columns) restricted epitopes in BALB/c and C57BL/6 mice, respectively; mean + SEM of 4 animals per group. The boxed inserts indicate the vaccines used to elicit the observed T cell responses. **b**) CD8+ T cell responses obtained 14 days post vaccination against NP derived H-2^d^ (left columns) and H-2^b^ (right columns) restricted epitopes. Mean +SEM of 4 animals per group. **c**) CD8+ T cell responses obtained 60 days post vaccination against GP derived epitopes in C57BL/6 mice. Mean +SEM of 7–8 animals per group pooled from two separate experiments. **d**) CD8+ T cell responses obtained 60 days post vaccination against NP derived epitopes in C57BL/6 mice. Mean +SEM of 7–8 animals per group pooled from two separate experiments. **e**) Weight curves of C57BL/6 mice vaccinated as in **c-d** and challenged with 10^6^ pfu of LCMV clone 13 60 days later. Mean weight +SEM of 12 mice until day 10 post infection and 8 mice thereafter. **f**) Spleen virus titers on day 10 post infection in mice vaccinated as in **c-d** and challenged with LCMV clone 13 60 days later. Points represent individual mice.

Despite the presence of two rather strong H-2^b^-restricted epitopes in the LCMV GP (GP33, GP276), numerous studies have confirmed a superior immunogenicity of the NP epitope, NP396, and a superior antiviral capacity of NP396 specific CD8+ T cells in H-2^b^ mice, such as C57BL/6 [17,18]. For this study we evaluated the vaccine primed responses induced by previously described adenoviral vaccines (Ad-) expressing LCMV GP and NP with (Ad-IiGP/Ad-IiNP) or without (Ad-GP/Ad-NP) covalent linkage to Ii [3]. Fourteen days after vaccination with 2x10^7^ IFU of either vector s.c. in the right hind foot, both NP encoding vaccines were found to induce robust CD8+ T cell responses not only in C57BL/6 (H-2^b^) mice, but also in BALB/c (H-2^d^) mice (Fig. 1b), whereas the CD8+ T cell responses elicited by the GP containing vaccines were highly dependent on Ii (P<0.05, comparing the sum of responses to all GP epitopes), and quantitatively superior in C57BL/6 as compared to BALB/c mice (Fig. 1a, P<0,05 comparing the sum of responses to all epitopes). These data not only confirmed that adenovirus expressing unlinked NP is more immunogenic than unmodified GP (P<0.05, comparing the sum of responses to all epitopes), but also that we could induce appreciable CD8 T cell responses above 10^6^ responding cells against both antigens in both strains of mice using the Ii linked vaccines (Fig. 1a-b). For subsequent immunization and challenge studies, we focused on Ad-GP, Ad-IiGP, Ad-NP and Ad-IiGP+Ad-NP vaccinations in C57BL/6 mice as we in this strain of mice could achieve the greatest similarity between the responses elicited by the Ad-IiGP and Ad-NP vaccines (see further below), although the Ad-IiGP response was somewhat lower at this early time-point. We refrained from attempting to reach similar response levels by adjusting the virus vector dosage as we had previously shown that vaccine dose may influence the phenotype of the elicited cells [19].

### Vaccine induced control of viral infection

To evaluate the efficiency of the selected vaccination regimens during the memory phase, we first vaccinated groups of C57BL/6 mice with Ad-GP, Ad-IiGP, Ad-NP or Ad-IiGP+Ad-NP, and 60 days later we enumerated CD8+ T cells capable of responding to each of 4 different GP or NP encoded epitopes by intracellular cytokine staining (ICS). The results confirmed that Ad-IiGP vaccination elicited a quantitatively superior (P<0.05, comparing the sum of responses to all GP epitopes) and broader CD8+ T cell response towards the GP epitopes than did Ad-GP (three out of four tested epitope specific responses consistently detected in Ad-IiGP vaccinated mice as compared to one out of four in mice given Ad-GP; P<0.05, Fig. 1c), and demonstrated that the Ad-NP vaccine induced a rather narrow, NP396 focused response(Fig. 1d). Combined Ad-IiGP+Ad-NP vaccination elicited a broad response to GP epitopes and an NP response focused towards NP396. At this time-point there was no quantitatively significant difference in the sum of measured responses towards Ad-IiGP and Ad-NP. To study the clinical effect of prior vaccination, mice immunized in the described manner were challenged 60 days later with 10^6^ pfu of LCMV clone 13 i.v. and monitored for signs of disease by evaluation of weight loss (Fig. 1e) and for virus control by determination of the infectious viral load in the spleen 10 days post infection (dpi) (Fig. 1f).

Unvaccinated mice showed a protracted weight loss and high viral loads in the spleen at 10 dpi, whereas both the Ad-IiGP, the Ad-NP and the Ad-IiGP+Ad-NP vaccinated mice had undetectable virus loads in the spleen and did not at any time display a weight loss (P<0.05 comparing unvaccinated mice to Ad-IiGP, the Ad-NP and the Ad-IiGP+Ad-NP vaccinated mice). The mice in the Ad-GP vaccinated group demonstrated an intermediate response pattern with suggestion of an accelerated, but transient and reduced weight loss (P<0.05 from day 10 post infection as compared to unvaccinated mice), and no infectious virus was detectable at 10 dpi. In previous studies from our group [3,20], we have found easily detectable infectious virus in Ad-GP vaccinated, LCMV clone 13 challenged mice at 5 dpi, but not at 10 dpi, indicating that virus control in these mice is established in the interval between 5–10 dpi, whereas the Ad-IiGP vaccinated mice had controlled the infection in the spleen already by 5 dpi [21]. It should be noted that although we can no longer detect virus in the spleen by standard plaque assays, the animals have not cleared the infection. Several months after infection of vaccinated animals we were able to detect infectious virus by transferring kidney homogenates into naïve recipients which subsequently seroconverted (data not shown).

### Infection induced CD8+ T cell responses

**Fig 2:**
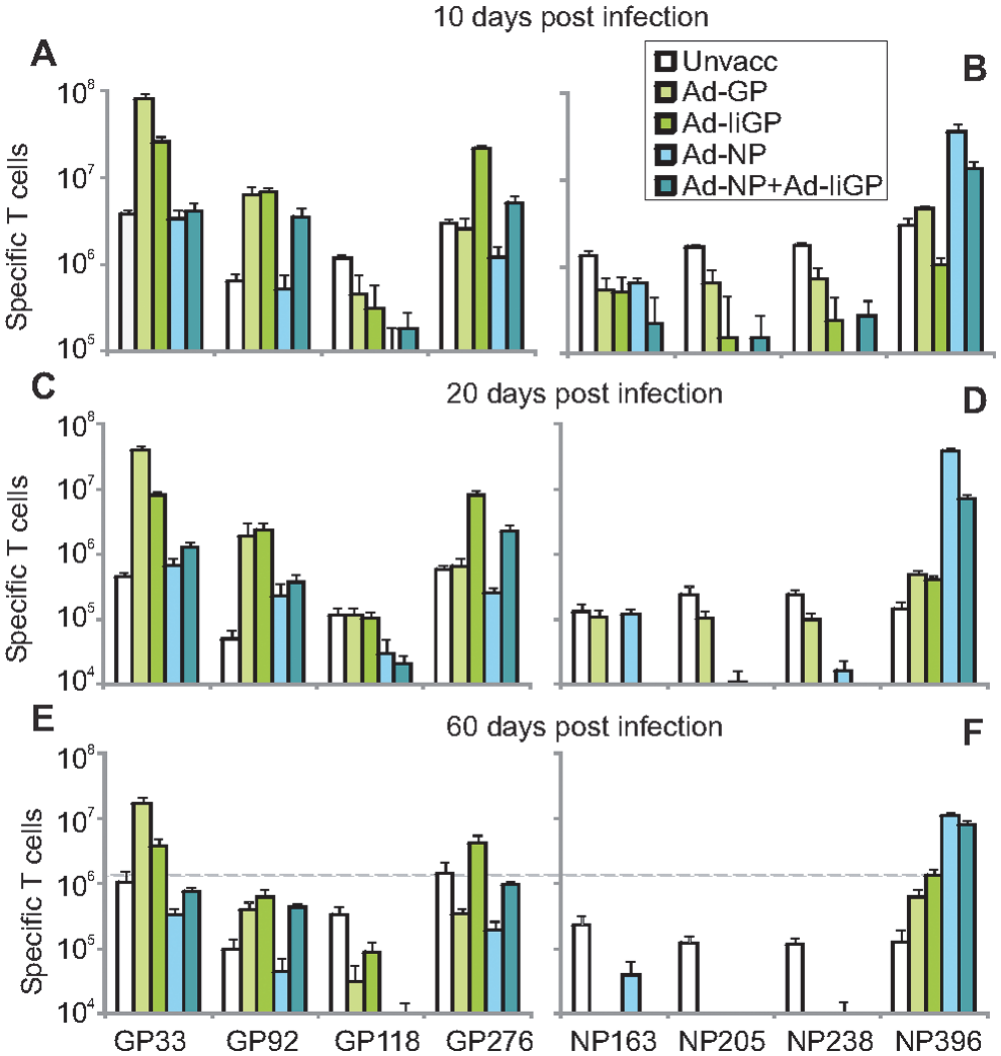
Vaccine and infection triggered CD8+ T cell responses. C57BL/6 mice were vaccinated with the adenoviral vaccines shown in the boxed insert and challenged with 10^6^ pfu of LCMV clone 13 60 days later. Spleens were harvested at 10 (**a-b**), 20 (**c-d**) and 60 (**e-f**) days post infection, and numbers of IFN-γ producing epitope specific CD8+ T cells were determined for GP (**a, c, e**) and NP epitopes (**b, d, f**). Mean +SEM of 7–8 mice per group pooled from two separate experiments. The dotted line in e-f shows the magnitude of the NP396 specific response achieved in Ad-IiGP vaccinated mice.

**Fig 3:**
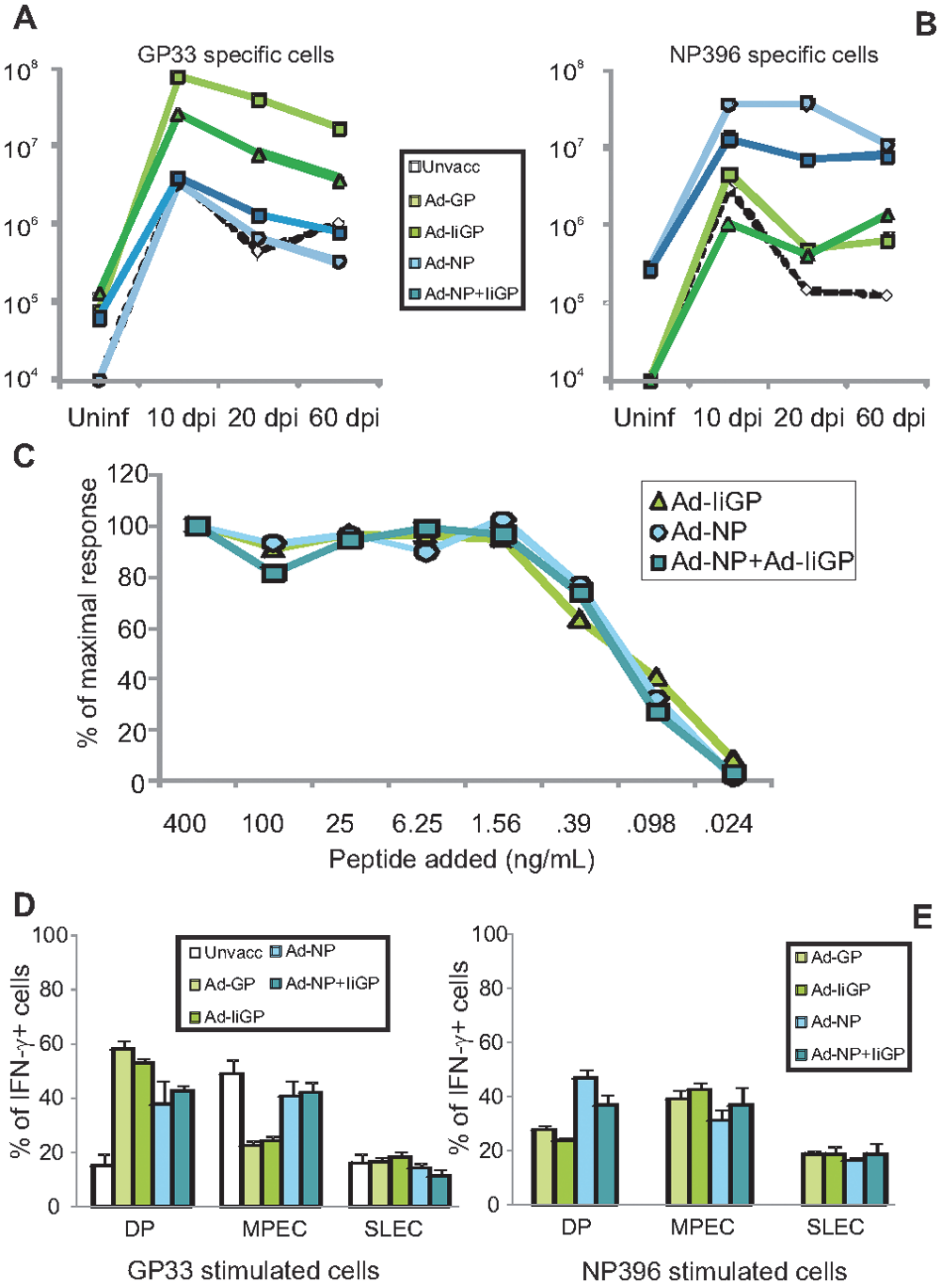
Numbers and phenotypes of memory CD8+ T cells. **a)** Numbers of GP33 specific CD8+ T cells from the mice described in Fig. 2 were plotted as a function of time. **b)** Numbers of NP396 specific CD8+ T cells from the mice described in Fig. 2 were plotted as a function of time. **c)** Pools of cells (4 mice per group) from the mice shown in Fig. 2e-f were stimulated with decreasing amounts of NP396. Results are depicted as the percentages of the response obtained at the highest peptide dose. Cells from similarly treated mice were also stimulated with GP33 or NP396 peptide, respectively, and the percentages of the CD8+ T cells producing interferon-γ that also expressed certain combinations of CD127 and KLRG-1 are shown, CD127+/KLRG1+ (DP), CD127+/KLRG1- (MPEC) and CD127-/KLRG1+ (SLEC)). **d**) Mice infected 60 days previously stimulated with GP33, e) mice infected 60 days previously stimulated with NP396 epitopes. Shown are mean +SEM of 4 mice per group.

**Fig 4:**
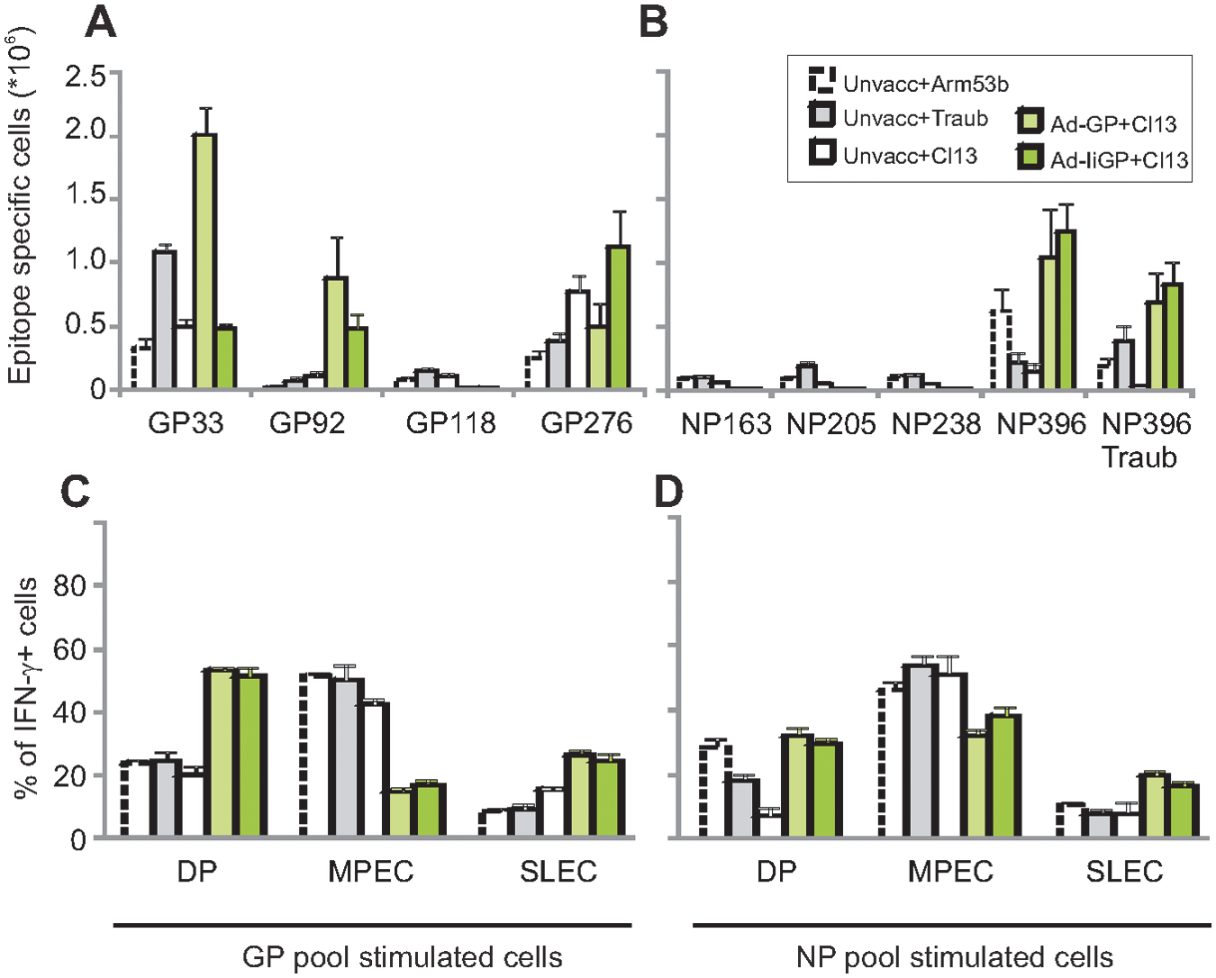
Comparison of vaccine attenuated Cl13 infection and primary infection with Cl13, Armstrong 53b or Traub strain of LCMV on the generation of memory CD8+ T cells. Cells from mice infected as indicated or vaccinated as indicated and then clone 13 challenged were stimulated with individual GP or NP peptides (**a-b**), or with a pool of GP (**c**) and NP (**d**) peptides 150 days after infection. The total number of splenic CD8+ T cells producing interferon-γ is shown in **a-b**. The percentage of cell producing interferon-γ in response to GP (**c**) or NP (**d**) peptides, that also expressed certain combinations of CD127 and KLRG-1 are depicted, CD127+/KLRG1+ (DP), CD127+/KLRG1- (MPEC) and CD127-/KLRG1+ (SLEC)). Shown are mean +SEM of 4–5 mice per group.

Functional CD8+ T cell-mediated immunity in vaccinated, virus challenged animals was evaluated by enumerating CD8+ T cells capable of responding to 4 different GP or NP encoded epitopes with IFN-γ production, 10 (Fig. 2a-b), 20 (Fig. 2c-d) and 60 (Fig. 2e-f) dpi. Compared to the pre-exposure values presented in Fig. 1c-d, this analysis not only revealed a massive expansion of CD8+ T cells specific for the vaccine encoded epitopes at early time points post infection, but also the appearance of virus primed responses towards epitope present in the challenge viruses, but not in the vaccine. To better appreciate the development of the responses we have plotted the kinetics of the CD8 T cell response to the immunodominant GP33 and NP396 specific epitopes in a separate figure (Fig. 3a-b). As would be expected, the CD8+ T-cell response towards the dominating vaccine encoded epitope reached very high levels and then declined following control of the acute phase of the infection. Interestingly, although both types of GP vaccinated mice demonstrated strong recall response against GP33 (~1000 fold increase for Ad-GP vs. ~200 fold increase for Ad-IiGP on day 10 post infection, and 228 vs. 29 fold on day 60 post infection, respectively), vaccination with Ad-NP and Ad-IiGP in combination resulted in much less pronounced expansion of GP33 specific CD8+ T cells as compared to that in Ad-GP or Ad-IiGP vaccinated mice (66 fold expansion on day 10 post infection and 5 fold on day 60 post infection. P<0.05 for either comparison). Conversely, fairly robust expansion of NP396 specific CD8+ T cell expansion was observed both in Ad-NP (~131 fold on day 10 post infection, 39 fold on day 60 post infection) and combined Ad-NP and Ad-IiGP vaccinated mice (~131 fold on day 10 post infection, 31 fold on day 60 post infection). When comparing GP and NP vaccinated mice, it was also clear that following viral challenge, the mice in the Ad-IiGP vaccinated groups raised a stronger virus primed response 60 days post infection against non-vaccine expressed NP396 than did NP vaccinated mice against any non-vaccine expressed GP epitope (P<0.001 comparing NP396 responses in Ad-IiGP vaccinated mice with GP33 responses in Ad-NP vaccinated mice). It may be noted that Ad-IiGP vaccinated mice also raised a stronger NP396 specific response 60 days post vaccination than did Ad-GP vaccinated animals despite lower GP33 expansion and overall a more rapidly controlled infection (P<0.05). However, this response pattern was not seen at later time points (Fig. 4b) and therefore might not be important in the long term. The early expansion of NP396 specific cells in Ad-GP vaccinated animals largely paralleled the expansion in unvaccinated animals with a minor reduction in Ad-IiGP vaccinated animals, but differed in two respects: NP396 specific cells from both GP vaccinated groups were protected from exhaustion between day 10 and day 20 pi, and the Ad-IiGP vaccinated group even exhibited an expansion of this population from day 20 to day 60 (Fig. 3b), while the GP specific response in both groups contracted during this time period (Fig. 3a) as did the NP specific response in NP vaccinated animals.

To see if these differences in the kinetics of NP396 specific responses were reflected in their quality, we evaluated the functional avidity of the cells specific for NP396 at 60 dpi by plotting the fraction of the maximal IFN-γ response induced by decreasing concentrations of specific peptide (Fig. 3c). The virus induced NP396 specific T cells in Ad-IiGP vaccinated and virus challenged mice were found to be as functionally avid as the vaccine primed and virus expanded NP396 specific CD8 T+ cells in previously Ad-NP or Ad-IiGP+Ad-NP vaccinated mice (Ad-GP vaccinated animals were not included in this analysis as too few NP396 specific cells were available for an accurate measurement). This indicate that vaccination with Ad-IiGP allows the highly avid T cell precursors directed against NP396 to persist during the acute phase of clone 13 infection, and then expand, likely as a result of their sensitivity to the low levels of persisting antigen during the chronic phase of the infection [22].Indeed, compared to GP specific responses, NP responses in all vaccinated groups tend to be slower to contract. This likely reflects a higher sensitivity of the latter cells for the limited antigenic stimulation associated with low grade chronic infection (see above) as previously documented in B-cell deficient mice [16]. Pathogen specific T cells present during controlled chronic infections not only display a hierarchy with regard to specificity and avidity, but may also differ with respect to phenotype [23]. To address this, we decided to characterize the cells responding at 60 dpi with respect to the expression of CD127 and KLRG-1, as several successful attempts have been made to correlate control of acute resolved infections, chronic infections, and latent infections with different expression patterns of these cell surface markers [24–27]. In the LCMV system, the phenotype of the virus-specific memory CD8+ T cells in different situations has been carefully characterized. Accordingly, memory cells generated in the context of an acutely resolved infection tend to be less terminally differentiated than T cells associated with persistent infection, and usually express higher levels of CD127 and lower levels of KLRG-1 [28,29]. Especially relevant during the contraction phase of the immune response, a nomenclature has been proposed where CD127+/KLRG-1- cells are referred to as memory precursor effector cells (MPECs) and generally represent the cells with the greatest capacity to develop into central memory cells [30], whereas CD127-/KLRG-1+ cells are termed short lived effector cells (SLECs) and are associated with acute or ongoing infection. Double positive (DP) cells seem to represent an intermediate phenotype whereas double negative (DN) cells are thought to represent early effectors that have not reached terminal differentiation [23]. Although it is not entirely established to which extent these functional attributes are maintained within the phenotypes following pathogen control or repeated immunizations, we and others have found they tend to represent a useful testament to the stimulation history preceding their enumeration in a memory response. Following a resolved acute infection CD127+/KLRG1- cells normally dominate the response, but varying numbers of DP cells also persist [28]. Such double positive cells can be stably expanded through repeated virus vectored immunizations [31]even though they were originally reported to have a reduced half-life compared to CD127+/KLRG- cells and tend to proliferate less in response to antigen and cytokine stimulation [13]. However, they maintain expression of cytotoxic molecules longer after pathogen clearance and can be considered effector memory cells [28,29].Accordingly, the T cells harvested at 60 dpi were co-stained for KLRG-1 and CD127, and the responding antigen specific T cells were subdivided into MPEC, SLEC and DP cells (Fig. 3d). Overall, vaccine primed and virus expanded CD8+ T cells tended to accumulate in the DP fraction, whereas virus primed T cells, present in the same individuals, more frequently had the phenotype of CD127+/KLRG-1- central memory cells (Fig. 3c-d). A notable exception was the NP396 response in the unvaccinated LCMV clone 13 infected mice, in which case too few cells were present to be relevantly subdivided. Our results are thus consistent with an imprinting on secondary responses with respect to a higher KLRG-1 expression (P<0.05, for GP33 specific and NP396 specific T cells when comparing Ad-IiGP and Ad-NP vaccinated animals respectively), which has previously been reported by several authors [11–13].

### Comparison of vaccine attenuated and Armstrong 53b, Clone 13 and Traub infections for induction of dominant antigen specific CD8+ T cell responses

Our initial analysis indicated that IiGP vaccinated mice rapidly control the LCMV clone 13 infection and still allowed residual virus to drive a substantial NP specific response. We therefore decided to compare the virus induced CD8+ T cells in vaccinated mice to those induced following a prototypic acute viral infection that is rapidly controlled without vaccination. Thus, at 150 dpi GP and NP specific T cells from mice vaccinated with either Ad-IiGP or Ad-GP and challenged with LCMV clone 13 were compared to those from unvaccinated mice infected with LCMV clone 13, Armstrong 53b or the Traub strain. At the doses used, these viruses forms a hierarchy in their ability to persist and suppress immune responses, with clone 13 being the most and Armstrong 53b the least persistent and immunosuppressive [32].

This analysis demonstrated that the subset of vaccine primed GP specific CD8+ T cells, which had undergone massive acute expansion in infected, vaccinated mice, contracted somewhat between 60 and 150 dpi (compare Fig. 2e and Fig. 4a; the fold contraction was 8.6 fold for Ad-GP vaccinated animals and 7.8 fold for Ad-IiGP vaccinated animals, respectively). In contrast, the virus primed CD8+ T cell response that had been established towards NP in the Ad-IiGP and, to a lesser extent, in Ad-GP vaccinated mice was quite stable (compare Fig. 2f and Fig. 4b, Ad-GP vaccinated animals had a 67% expansion and Ad-IiGP vaccinated animals had a 7% reduction of their NP396 specific T cell numbers). Rather impressively, in the long-term, virus primed NP396 responses in the Ad-IiGP vaccinated mice numerically surpassed the responses seen after natural and self-limiting infection with the LCMV strains Traub (P<0.01) and Armstrong 53b (P<0.05)(Fig. 4b).

As the Armstrong clones 13 and 53b viruses encode a NP396 epitope, which differs from that expressed by the Traub strain, we decided to evaluate the cross-reactive potential of the NP396 specific T cells present at 150 dpi. Comparison of the responses towards the two epitope variants revealed that NP396 specific cells from GP vaccinated, clone 13 challenged mice matched the cells from Armstrong 53b infected mice in this respect, and thus the virus primed CD8+ T cells in vaccinated mice are likely to be as good in recognizing sequence diverted viral strains as those raised by a self-limiting natural infection.

The phenotypes of GP and NP specific cells analyzed 150 dpi were rather similar to those observed at 60 dpi, and, importantly, resembled those of Armstrong 53b induced T cells with regard to the NP specific subset, but not the GP specific subset, where the KLRG-1 expression was substantially higher in GP vaccinated mice (Fig. 4c-d, P<0.05 comparing either Ad-GP or Ad-IiGP vaccinated and clone 13 challenged mice with Armstrong 53b infected mice). Again, these data confirm an importance of stimulation history on the resulting T-cell phenotype, in this case irrespectively of whether virus control requires prior vaccination or because the virus strain by its nature induces a self-limiting infection.

### The effect of vaccine antigen on the long-term CD8+ T cell response

**Fig 5:**
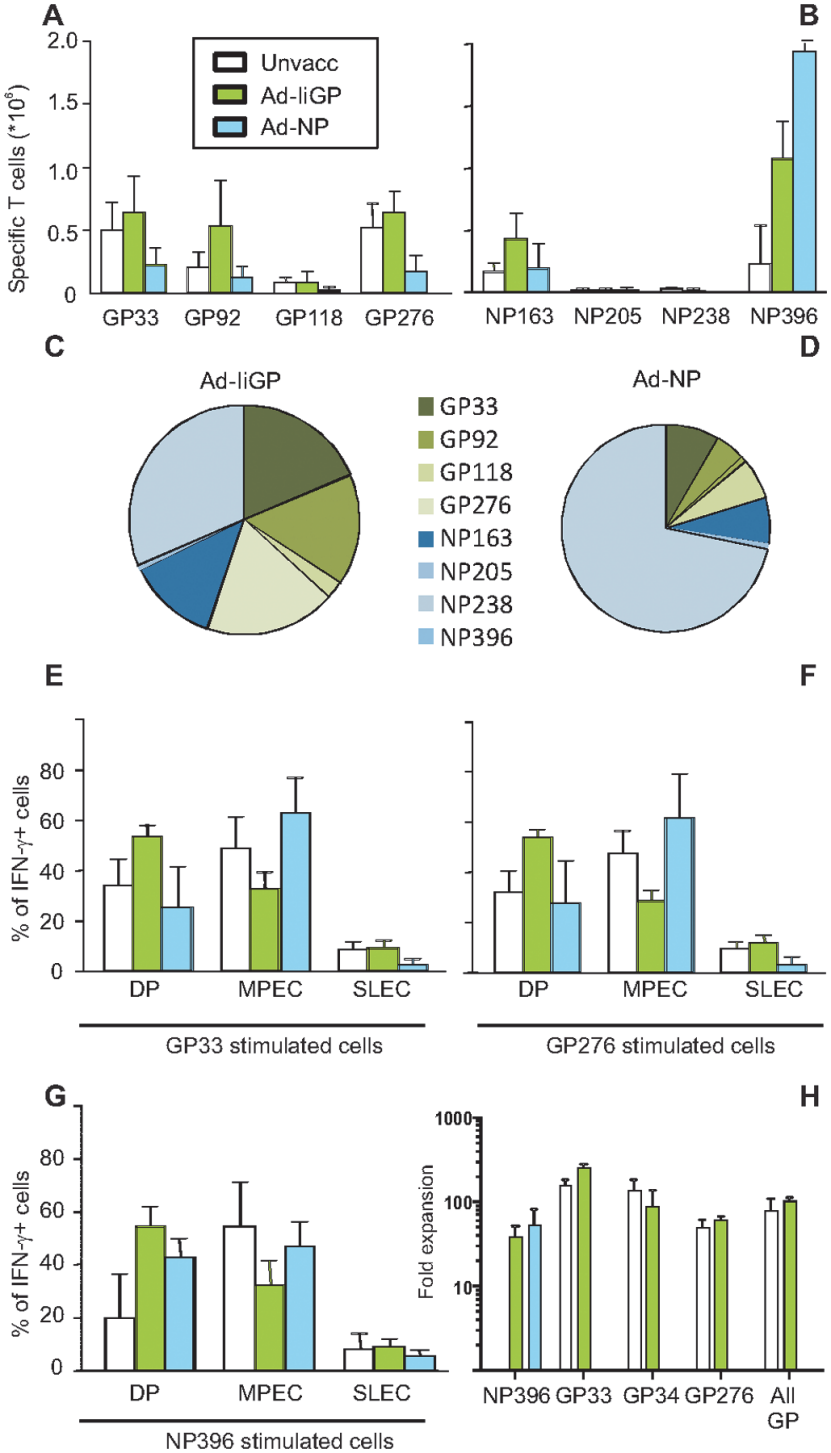
Long term immune responses, phenotype and secondary in vivo expansion. Mice were either infected with LCMV clone 13 or Ad-IiGP or Ad-NP vaccinated and then challenged with LCMV clone 13 as indicated. One year after LCMV clone 13 infection, mice were sacrificed and spleens were harvested for characterization of the CD8+ T cell response. **a-b**) Enumeration of CD8+ T cells responding to specific GP (**a**) or NP (**b**) peptide stimulation with interferon-γ production. **c-d**) Fraction of CD8+ T cells from Ad-IiGP (**c**) and Ad-NP (**d**) vaccinated mice responding to individual specific peptides as a fraction of total numbers of GP plus NP specific cells. The small difference in the sizes of the pie charts reflects the difference in the averages of total numbers of cells recognizing GP and NP epitopes in the two groups. **e-g**) phenotype of responding cells. The percentage of cell producing interferon-γ in response to GP33 (**e**), GP276 (**f**) or NP396 (**g**) peptide, that also expressed certain combinations of CD127 and KLRG-1 are depicted, CD127+/KLRG1+ (DP), CD127+/KLRG1- (MPEC) and CD127-/KLRG1+ (SLEC)). Shown are mean + SEM for 8 animals per group. **h**) Expansion following vaccinia GP or vaccinia NP challenge. 10.000 GP33+GP34+GP276 or 10.000 NP396 specific cells pooled from the animals described above (CD45.2+) were transferred to naïve recipients (CD45.1+), and these were challenged the following day with vaccinia expressing NP (NP396) or GP (GP33, GP34, GP276 and GP pool). Fold expansion is the number of donor T cells quantified by the indicated tetramer in vaccinia infected recipients as compared to the number detectable in unchallenged recipients. Shown are mean +SEM of 5 recipient mice per group.

CD8+ T cell responses which should successfully control infections with pathogens capable of establishing a chronic state of infection, e.g. HIV, will need to be effective not in months, but rather years after infection. We therefore decided to analyse T cell responses in 16 months old mice previously vaccinated with Ad-IiGP, Ad-NP or left unvaccinated at 2 months of age, and infected with LCMV clone 13 at 4 months of age (Fig. 5). In this cohort we confirmed the superior ability of Ad-IiGP vaccinated mice to raise responses towards NP epitopes (Fig. 5a) as compared to induction of GP specific responses in Ad-NP vaccinated mice (Fig. 5b)(P<0.05 comparing the sum of responses). Yet, small differences are observed, and compared to earlier time-points we did see modest GP responses in NP vaccinated mice (Fig. 5a), and a response towards NP163 in all groups (Fig. 5b) not seen at 60 days post infection (Fig. 2f). In order to clearly appreciate the impact of vaccine strategy on the breadth of the induced responses, we also plotted the mean response of Ad-IiGP (Fig. 5c) and Ad-NP (Fig. 5d) vaccinated mice as a fraction of the total GP and NP specific responses. A more diverse T cell response appears evident in Ad-IiGP vaccinated and LCMV challenged as compared to those mice vaccinated with Ad-NP before challenge. Notably, the sum of all measured GP and NP responses is also on average 23% higher in those animals vaccinated with Ad-IiGP compared to Ad-NP (2.76 vs. 2.16 million specific splenocytes, respectively)). Looking at KLRG1 and CD127 expression important differences were observed. Thus, even though the GP33 and GP276 specific responses still bore evidence of prior vaccine exposure in the Ad-IiGP vaccinated animals, with a higher frequency of DP cells and less CD127+/KLRG1- cells (P<0.001 for both comparisons), the NP396 specific cells were no longer phenotypically different between the Ad-IiGP and Ad-NP vaccinated animals (Fig. 5e-g). If anything, the Ad-NP vaccinated mice had more NP396 specific MPECs and fewer DP cells than the Ad-IiGP primed mice (the differences were small, but significant, P<0.05).

Previous studies addressing the impact of repeated antigen exposure on T cell functionality have revealed that the ability to undergo secondary expansion is reduced in repetitively stimulated cells, but this have always been paralleled by phenotypic differences including high KLRG1 expression [11,13]. To study this aspect further, we also performed an analysis of the per-cell ability of NP or GP specific cells to expand in response to secondary challenge. This was done by transferring equal numbers of primed antigen specific T cells from CD45.2+ donors to naïve CD45.1+ hosts followed by challenge of the recipients with vaccinia virus expressing GP or NP; for control some recipients were left unchallenged. Six days later the expansion of the transferred cells was calculated by enumerating tetramer specific T cells in the recipient spleens (Fig. 5h).

This analysis revealed a roughly similar expansion of all the transferred cell specificities, irrespectively of the first immunization except for the GP33 specific T cells, which expanded slightly, but significantly, less when coming from unvaccinated mice than from Ad-IiGP vaccinated mice (P<0.05). The differences between different NP specific T cell populations were not statistically significant. Thus, the phenotypic imprinting associated with prior vaccination appears to be lost over time with respect to the immunodominant NP396 epitope, but not the tested GP epitopes.

## Discussion

In this report we have studied the advantages and disadvantages of using prophylactic vaccines targeting subdominant rather than immunodomant epitopes, and the study highlights two primary findings, which are relevant in the context of planning future vaccine stategies. The first finding is that efficient vaccination with non-dominant antigens can be used as a means to select for the ability during subsequent viral challenge to raise primary responses towards immunodominant antigens and thus increase the breadth of the T-cell response. In contrast, vaccine targeting of immunodominant antigens was found to inhibit virus-induced responses against non-dominant antigens during an acutely controlled, but persistent infection. The second important finding of this study is that the phenotypic traits and reported proliferative impairment of CD8+ T cells associated with repeated antigenic stimulation, at least with respect to the immunodominant NP epitope, were not stable over time at least in the splenic compartment.

The latter finding is important as repeated antigenic stimulation has been shown to markedly impact the quality of the generated memory T cells including their phenotype [13,33] and the ability to control a chronic infection and undergo secondary expansion [11]. In our set-up, the cells first primed in the context of the viral challenge represent primary cells, and the cells already primed during vaccination are undergoing what equals to a secondary response during the viral infection. When comparing primary and secondary responding T cells, our phenotypic analysis showed the expected impact of sequential immunization per se [13], and of the human serotype 5 adenoviral priming [34,35], in particular at the intermediate time point 60 days after virus infection. If the phenotypic changes associated with sequential immunization had been permanently imprinted on the involved T cell populations, it would have had grave implications for vaccination concepts aiming at inducing the strongest possible response against the most critical viral antigens for prophylaxis against chronic infections. However, this is not what we found. On the contrary, proliferation-proficient T cells reacting against immunodominant NP antigens with a high proportion of CD127+/KLRG1- T cells can be generated not only *de novo* after Ad-IiGP vaccination, but also late in the NP vaccinated mice. Ad-GP vaccination was also less efficient in promoting NP specific responses. These findings suggest that driving essential T cell populations towards an effecter memory phenotype may not harm their long term proliferative potential during a chronic infection, but that a broader overall response can be obtained by targeting sub-dominant epitopes. Regardless of the antigen chosen, we found that the important attribute of a vaccine to induce sustainable long-term responses, is the ability of that vaccine to secure a state of profound acute control of the viral infection. Adjuvant enhanced adenoviral vectors as we have used, heterologous virus vectored prime-boost immunization regimens, or persisting vectors may be well suited for this task [1,36–38]. The mechanistic basis for a split into a more stable phenotype regarding GP specific T cells, and phenotypic recovery of NP396 specific cells is not clear, but most likely reflects a continuous stimulation of the highly avid NP396 specific T cells but not of the GP specific cells during an infection that is kept at a very low level. Precedent for this distinction in responsiveness of GP vs NP specific cells can be found in the literature [16], and may also explain the more delayed pattern of contraction regarding NP specific responses as compared to GP responses in all vaccine groups. Whereas the long-term phenotype of the GP specific response are in agreement with newer reports supporting an ability of KLRG-1 effector memory phenotype cells to be maintained over time [39,40], the specific phenotypic recovery of the NP specific cells during a persistent chronic infection has not been reported before in the LCMV system. It is possible that the secondary memory NP396-specific cells are gradually replaced by primary responders derived from recent thymic emigrants as has previously been reported to take place during certain chronic viral infections [41], yet in the murine γ-herpesvirus model of chronic infection such a phenomenon does not seem to be necessary to explain a similar finding [42]. Alternatively, the continuous stimulation in the context of a very low viral load may facilitate a functional recovery simply by superior replicative capacity under competitive conditions of available cells with a central memory phenotype. Such an interpretation leads to the suggestion that previously reported differentiation of effector memory T cells into central memory phenotype cells may be driven by a low level of persisting antigen rather than being T cell intrinsic [43]. That such observations have been made with GP specific T cells might reflect the use of TCR transgenic cells with unnaturally highly avid TCRs [43]. Importantly for ongoing plans to develop adenoviral vectors for vaccination, irrespectively of the actual cause of the functional and phenotypic recovery of the NP396 specific T cells, we find no evidence suggesting that adenovector immunization negatively impact the long-term persistence and functionality of CD8+ T cells during a persistent infection as has been proposed [31,34,35,44].

Although high-quality CD8+ T cells specific for immunodominant epitopes can recover from repeated stimulation during a chronic infection there are other potential drawbacks from relying on such responses to which we here offer a solution. Most importantly, we find that using immunodominant antigens in the vaccine largely restricts the immune response to vaccine encoded specificities. Thus, using the NP vaccine alone induced a relatively narrow response highly focused on the immunodominant NP396 epitope, whereas using the Ad-IiGP vaccine enabled a broader GP specific response as well as a virus induced NP specific response. Using the vaccines in combination enabled induction of both GP and NP specific responses, but the anamnestic response against GP which followed viral challenge was reduced. This suggests that leaving out dominant epitopes may have advantages over combining vaccine antigens in regard to establishing the broadest possible response during the chronic phase of an infection. Whether or not such an increase in breadth will materialize into improved antiviral efficacy is not clear. The advantage will be counteracted by the delay in raising the most protective immunodominant antigen specific T cell response, yet magnified by putting increased selective pressure on the non-immunodominant T cell epitopes during early phases of the infection. Indeed, during HIV infection, early immunodominance was found to be the most important predictor of viral escape by mutation [45]. Unfortunately, such mechanisms are hard to address and validate in the LCMV system where chronic control, unlike in HIV, can be achieved by single-epitope specific vaccines [20,46].

In summary, we here present a strategy for broadening CD8+ T-cell responses towards dominant and non-dominant epitopes in relation to vaccination against chronic viral infections. We also put forward encouraging evidence that prior adenoviral vaccination may not negatively impact the long-term and protective immune response induced and maintained during a vaccine-attenuated chronic viral infection. The latter observation is consistent with observations made in HIV elite controllers where the most antigen sensitive cells are also among the most functionally capable [47], whereas based on earlier mechanistic studies involving TCR transgenic mice, they should have been the most exhausted [48–50].
